# Preparation of PVDF/PAR Composites with Piezoelectric Properties by Post-Treatment

**DOI:** 10.3390/polym10121333

**Published:** 2018-12-03

**Authors:** Woo Jin Oh, Hyeon Soo Lim, Jong Sung Won, Seung Goo Lee

**Affiliations:** Department of Advanced Organic Materials & Textile Engineering, Chungnam National University, Daejeon 305-764, Korea; woojin819@cnu.ac.kr (W.J.O.); hyeonsoo@cnu.ac.kr (H.S.L.); jswon20@cnu.ac.kr (J.S.W.)

**Keywords:** PVDF, piezoelectric, island-in-a-sea fiber, conjugate spinning, polyarylate, thermoplastic composite, drawing, poling

## Abstract

Thermoplastic composites were prepared using poly (vinylidene fluoride) (PVDF) as the matrix with piezoelectric properties and aromatic polyarylate (PAR) as the reinforcing component. The PVDF/PAR conjugate fibers were prepared by melt conjugate spinning. The PVDF/PAR composites were prepared by compression molding of the PVDF/PAR conjugate fiber laminates at various molding temperatures. Drawing and poling post-treatments of the PVDF/PAR composites were performed to increase the *β* crystalline phase content of the PVDF. The morphologies of the PVDF/PAR composites were observed by scanning electron microscopy, and the tensile properties were tested using an universal testing machine. The crystal structure of the PVDF/PAR composites was confirmed by Fourier transform infrared spectroscopy and X-ray diffraction. The piezoelectric properties were tested using voltmeters and multimeters. The post-treatments enhanced the content of the *β* crystalline phase of the PVDF matrix, thereby improving the piezoelectric properties of the composites. A molding temperature of 180 °C, drawing temperature of 90 °C, and poling voltage of 12 kV were identified as the optimal conditions for the preparation of the PVDF/PAR composite.

## 1. Introduction

Poly(vinylidene fluoride) (PVDF) is a semi-crystalline polymer that has been widely investigated for its piezoelectric properties and moderately good chemical resistance [1]. PVDF exists in four different crystal forms: *α*, *β*, *γ*, and *δ*. Since the *α* crystalline phase is monoclinic characterized by trans-gauche-trans-gauche (TGTG) conformation of molecular chains, the fluorine atoms are arranged antiparallel to the carbon main chain. Thus, the *α* crystalline phase does not exhibit spontaneous polarization and is non-polar. The *β* crystalline phase has an all-trans (TTTT) conformation with parallel arrangement of molecular chains [2,3]. Therefore, all the permanent dipoles are arranged in one direction, causing the *β* crystalline phase to exhibit the large spontaneous polarization. Piezoelectricity and ferroelectricity of PVDF are derived from the *β* phase [4]. The *α* phase is the most abundant and stable polymorph of PVDF. However, the *α* crystalline phase can be transformed into the *β* crystalline phase through drawing and poling treatments. These post-treatments can increase the content of the *β* crystalline phase and improve the piezoelectric properties of PVDF [5,6,7]. 

In general, studies related to PVDF as a piezoelectric material have mainly focused on a film type material [8,9]. However, film-type PVDF has poor mechanical properties for structural applications such as the skeleton structure or the parts of the robot. A certain kind of reinforcing is essential for enhancing the mechanical and structural properties of the PVDF [10]. 

Polyarylate (PAR) is a liquid crystalline aromatic polymer with repeating molecular units consist of ester groups (–CO–O–) and aromatic rings. Given its excellent mechanical properties and chemical stability, PAR has been used as a reinforcing material in a variety of applications including automobiles, electronic displays, and protective coverings [11,12]. Fiber-type PAR shows high structural orientation and fibril structure suitable for composite reinforcement [13]. For this reason, we tried to develop a thermoplastic composite using PVDF as the functional matrix and aromatic PAR as the reinforcement.

Thermoplastic composites have various advantages including high-speed processability and recyclability, besides being environmentally friendly [14]. Therefore, many studies on thermoplastic composite have been completed [15,16]. However, since the matrix resin (PVDF) of thermoplastic composite has a high melt viscosity, the resin is hardly impregnated into the fibers (PAR) [14]. To overcome this disadvantage, we used a conjugate fiber process (co-spinning) called the “island-in-a sea fiber method”. Through this process, island-in-a sea fibers consisting of two polymers (island-PAR and sea-PVDF) were prepared without impregnation problems. 

In this study, thermoplastic composites using PVDF as the matrix and PAR as the reinforcement (PVDF/PAR) were prepared using the PVDF/PAR island-in-a-sea fibers via compression molding at different molding temperatures. The composites prepared at the optimal condition were post-treated by drawing and poling. As a result of the post-treatment, the content of *β* crystalline phase of PVDF in the composite increased, which significantly enhanced the piezoelectric properties of the PVDF/PAR composite.

## 2. Materials and Methods 

### 2.1. Melt Spinning of Island-In-A-Sea Fiber

**Figure 1:**
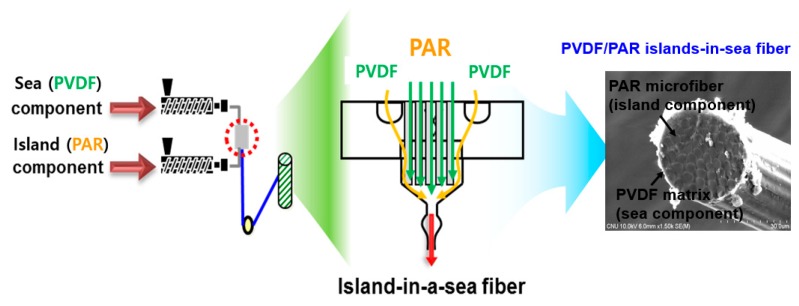
Schematics of the conjugate spinning process for the preparation of PVDF/PAR island-in-a-sea fiber.

The materials used in this study were PVDF (Kynar 720, Arkema, Colombes, France) and PAR (Vectra A950, Celanese, Irving, TX, USA). The PVDF/PAR island-in-a-sea fibers were prepared by melt conjugate spinning at 270 °C as shown as Figure 1. The conjugate spinning conditions are displayed in Table 1. PVDF and PAR were used as a sea component and the island component, respectively, in a weight ratio of 5:5. In this spinning system, two connected extruders for two polymers and a conjugate spinning nozzle with many sub-holes were used. The conjugated spun fibers were wound at a speed of 650 m/min. The diameter of the conjugated fiber and the PAR component in the fiber were 39 and 4.2 µm, respectively.

### 2.2. Preparation of PVDF/PAR Composites

**Figure 2:**
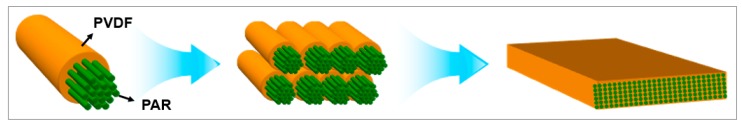
Schematics of the molding process of the PVDF/PAR island-in-a-sea fiber composite.

The PVDF/PAR composites were prepared by a compression molding (Figure 2). First, the PVDF/PAR island-in-a-sea fibers were wound, and prepregs were prepared by compression molding at 170 °C and 500 psi for 30 s. Thereafter, a couple of prepregs were laminated to prepare a composite material. The molding temperature *T_m_* based on the melting of PVDF matrix (174 °C) was set to 170, 175, 180, and 185 °C, and the molding time and pressure were fixed at 3 min and 1500 psi, respectively.

### 2.3. Post-Treatment

**Figure 3:**
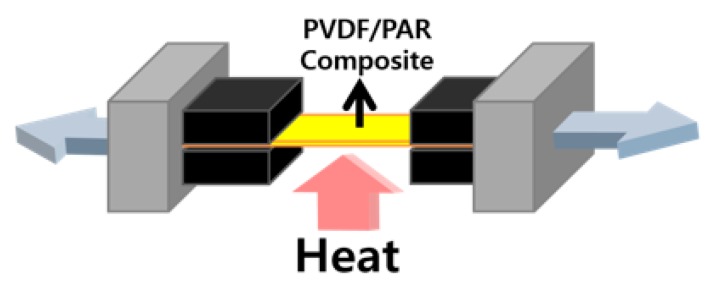
Schematics of PVDF/PAR composite drawing process.

**Figure 4:**
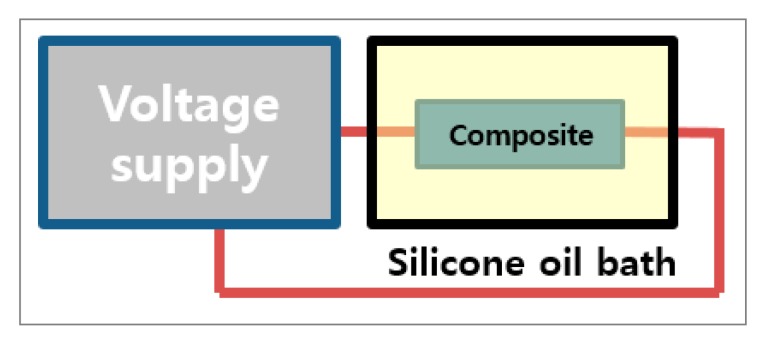
Schematics of PVDF/PAR composite poling process.

To increase the content of *β* crystalline phase of PVDF, drawing and poling post-treatments of the PVDF/PAR composites were carried out. The composites were prepared by compression molding at 180 °C and 1500 psi for 3 min. The drawing treatment was carried out in the axial direction of the PAR fiber (Figure 3). The drawing temperature was set to 70, 90, 110, and 130 °C, and the drawing ratio was fixed at 2.0. The poling treatment was carried out using a direct current (DC) supply (Figure 4). Prior to the poling process, silver paste was applied at both ends of the composite. The poling treatment was carried out for 3 h in a silicone oil bath at 105 °C, the Curie temperature of PVDF. The poling voltage was set to 6, 8, 10, and 12 kV.

### 2.4. Characterization

The microstructure of the PVDF/PAR composites was examined at 10 kV by scanning electron microscopy (SEM; S-4800, Hitachi, Tokyo, Japan). The tensile properties were measured using a universal testing machine (UTM; Model 4467, Instron, Norwood, MA, USA). Fourier transform infrared spectroscopy (FT-IR) in the frequency range from 1000 to 400^−1^ (iS50, Nicolet, Glendale, WI, USA) and X-ray diffraction (XRD) operated at 30 kV, 20 mA (XD/MAX-2200 Ultima/PC, Rigaku, Tokyo, Japan) were used to analyze the crystal structure of the composite. The piezoelectric properties of the PVDF/PAR composites were tested using voltmeters and multimeters (DMM7510, Keithley, Cleveland, OH, USA).

## 3. Results

### 3.1. Morphologies

**Figure 5:**
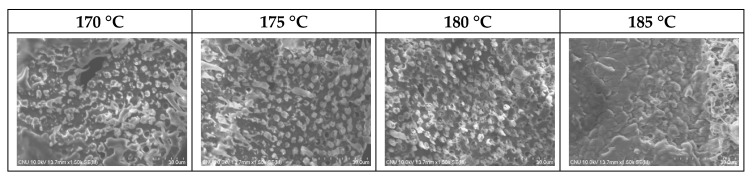
Cross-sectional SEM images of the PVDF/PAR composites prepared at different molding temperatures.

The cross-sections of the PVDF/PAR composites, prepared at various molding temperatures, were observed by SEM (Figure 5). The PVDF sea component was not completely melted at molding temperatures of 170 and 175 °C, whereas at 185 °C, the alignment of the PAR islands in the composite was disrupted by the high flowability of the PVDF sea component. Relatively good bonding among the island-in-a-sea fibers as well as good alignment of PAR islands were attained at a molding temperature of 180 °C.

### 3.2. Tensile Properties

**Figure 6:**
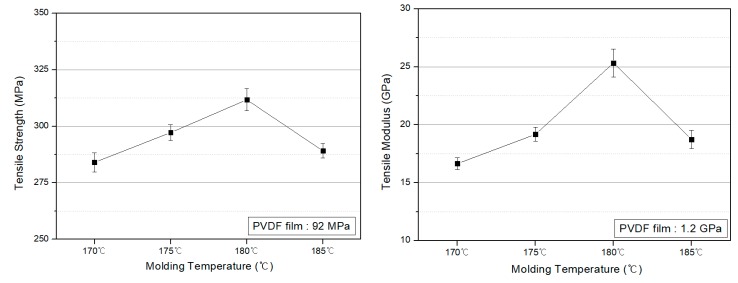
Tensile strength and tensile modulus of the PVDF/PAR composites prepared at different molding temperatures.

The tensile strength and modulus of the PVDF/PAR composites molded at temperatures below 180 °C were considerably low (Figure 6). This behavior is attributed to the incomplete melting of the sea component and the consequent formation of pores inside the composite that acted as defects. The PVDF/PAR composites prepared at 180 °C exhibited the maximum tensile strength and modulus. At 185 °C, the disruption in the arrangement of the PAR island component resulted in a decrease in the tensile strength and modulus.

Compared to the tensile strength and modulus of the PVDF film, which were 92 MPa and 1.2 GPa, respectively, the PVDF/PAR composites possess 3 and 20 times higher values, respectively. Therefore, the aim of this study—improving the mechanical properties of PVDF materials—could be achieved, thereby enabling their use in higher-level structural applications. Even though the content of PVDF is only 50% in the composite, which may reduce the piezoelectricity of the composite to some extent compared to that of the PVDF, the improvement in mechanical properties of the composite is important for its structural applications.

### 3.3. Crystal Structures and Properties

#### 3.3.1. FT-IR Analysis

**Figure 7:**
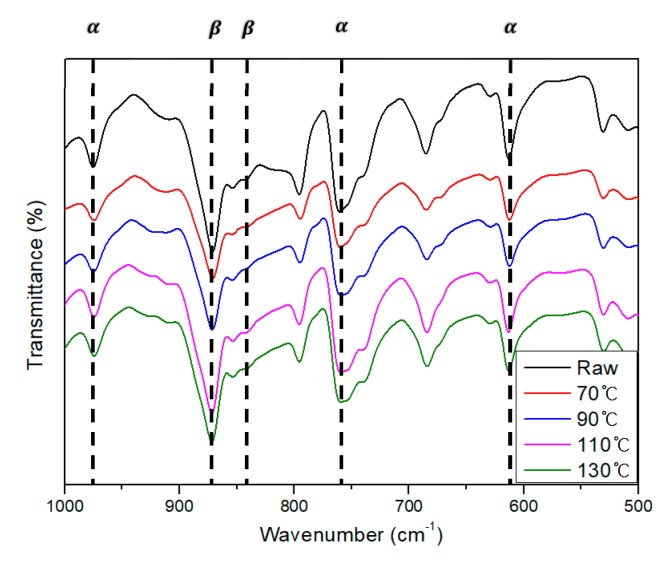
FT-IR spectra of the PVDF/PAR composites drawn at different temperatures.

**Figure 8:**
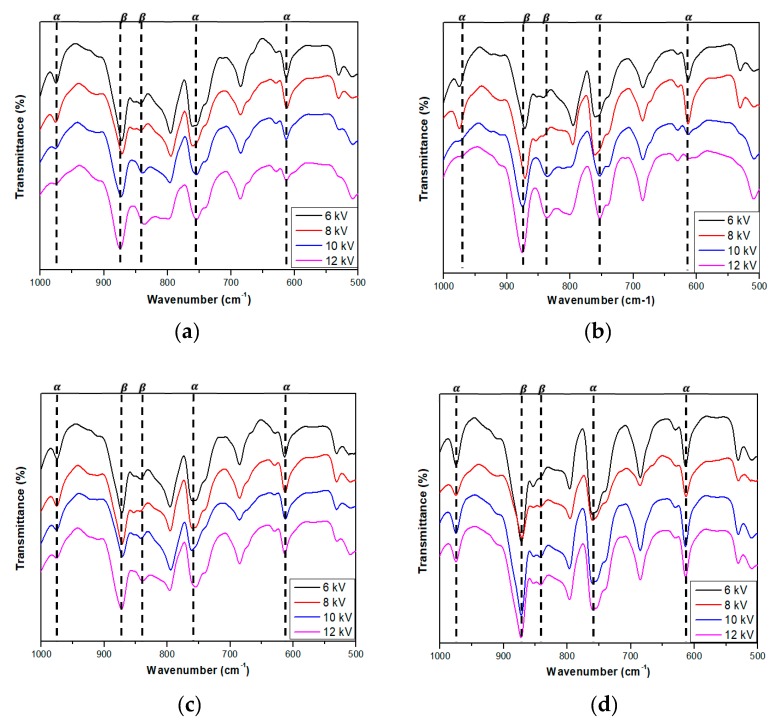
FT-IR spectra of the poled PVDF/PAR composites at drawing temperatures of (**a**) 70, (**b**) 90, (**c**) 110, and (**d**) 130 °C.

FT-IR results of the PVDF/PAR composites showed changes in the crystalline phase as a result of the drawing process (Figure 7). The bands observed at 976, 765, and 613 cm^−1^ in the FT-IR spectra can be attributed to the α crystalline phase, whereas the bands at 878 and 848 cm^−1^, to the *β* crystalline phase [6,7,17]. At drawing temperatures of 70 and 90 °C, the intensity of *β* crystalline phase increased and that of *α* crystalline phase decreased. At drawing temperatures of 110 and 130 °C, the intensity of *β* crystalline phase decreased and that of *α* crystalline phase increased. This is due to the *β* → *α* crystalline phase transition occurring above the Curie temperature of PVDF (103 °C). After the drawing process, the poling process was carried out at different voltage conditions and Figure 8 shows the results of the poling process. In Figure 8, the ratio of *β* crystalline phase increases significantly with increasing applied voltage during the poling process.

Based on the results of the FT-IR analysis, the relative intensities of the *β* crystalline phase were calculated using Equation (3) derived from the Beer-Lambert law shown in Equations (1) and (2). Here, *A_α_* and *A_β_* are the absorbances of the *α* crystalline phase and the *β* one, respectively; *L* is the thickness of the specimen; *C* is the average monomer concentration; *I*_0_ and *I* are the intensities of incident and transmitted light, respectively; *K* is the absorption coefficient at each wave number, and the values of *K_α_* and *K_β_* are 6.1 × 10^4^ and 7.7 × 10^4^ cm^2^/mol, respectively; *X* represents the degree of crystallinity of each phase; and *F*(*β*) represents the relative intensity of the *β* crystalline phase.

(1) $$A_{\alpha }=\log\left(\frac{I_{\alpha }^{0}}{I_{\alpha }}\right)=C\cdot K_{\alpha }\cdot X_{\alpha }\cdot L$$

(2) $$A_{\beta }=\log\left(\frac{I_{\beta }^{0}}{I_{\beta }}\right)=C\cdot K_{\beta }\cdot X_{\beta }\cdot L$$

(3) $$F\left(\beta \right)=\frac{X_{\beta }}{X_{\alpha }+X_{\beta }}=\frac{A_{\beta }}{\left(\frac{K_{\beta }}{K_{\alpha }}\right)A_{\alpha }+A_{\beta }}=\frac{A_{\beta }}{1.26A_{\alpha }+A_{\beta }}$$

**Figure 9:**
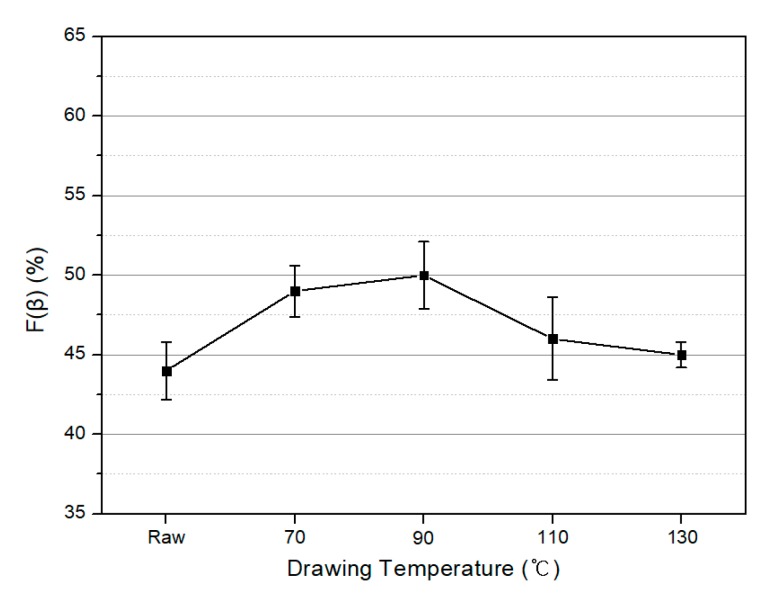
Relative intensity of the *β* crystalline phase in the PVDF/PAR composites with drawing temperature (Unpoled).

**Figure 10:**
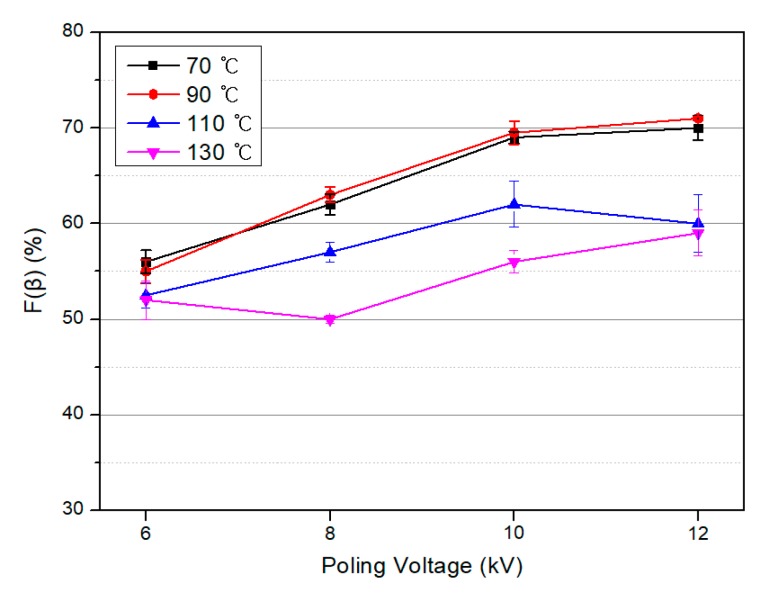
Relative intensity of the *β* crystalline phase in the PVDF/PAR composites as a function of the poling voltage.

The variation in the *β*-phase content of the PVDF/PAR composite *F*(*β*) with the drawing temperature and the poling voltage are shown in Figure 9 and Figure 10, respectively. As shown in Figure 9, *F*(*β*) increases at drawing temperatures of 70 and 90 °C, which are lower than the Curie temperature of PVDF, and shows the largest value of 50% at 90 °C. Figure 10 illustrates the results of *F*(*β*) at different poling voltages after the drawing process. As the poling voltage increases, the dipoles are arranged in one direction, and the *F*(*β*) values increase. It is evident that *F*(*β*) is more affected by the poling voltage than the drawing temperature. We concluded that the maximum value of *F*(*β*) is achieved at a drawing temperature of 90 °C and a poling voltage of 12 kV, and the maximum value of *F*(*β*) was calculated to be 71%.

#### 3.3.2. X-ray Diffraction (XRD) Analysis

**Figure 11:**
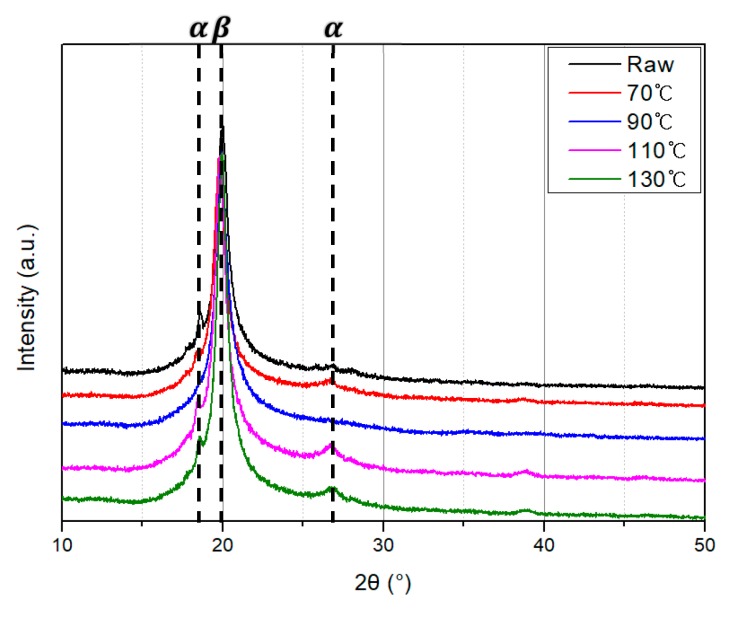
XRD patterns of the PVDF/PAR composite as drawing temperature (Unpoled).

**Figure 12:**
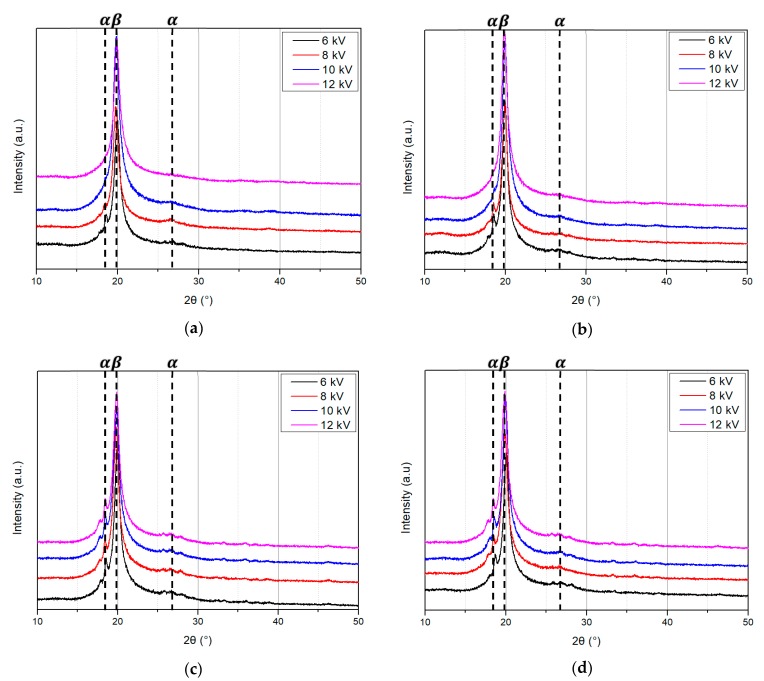
XRD patterns of the poled PVDF/PAR composites at drawing temperature of (**a**) 70, (**b**) 90, (**c**) 110, and (**d**) 130 °C.

Figure 11 and Figure 12 show the XRD patterns of the PVDF/PAR composite prepared at different drawing and poling conditions, respectively. The peaks at 2θ values of 18.4 and 26.5° can be assigned to the *α*(100) and *α*(020) reflections of the α phase of PVDF, respectively. The peak observed at 20.3° is assigned to *β*(110) reflection of *β* crystalline phase [18,19]. The intensity of the *β*-phase peak of the PVDF/PAR composite poled at 12 kV after drawing at 90 °C was the highest among all the samples. This result about the intensity of the *β*-phase is very similar to the results of FT-IR analysis. The increase in intensity of *β*-phase with increasing poling voltage is shown in Figure 12.

### 3.4. Piezoelectric Properties

**Figure 13:**
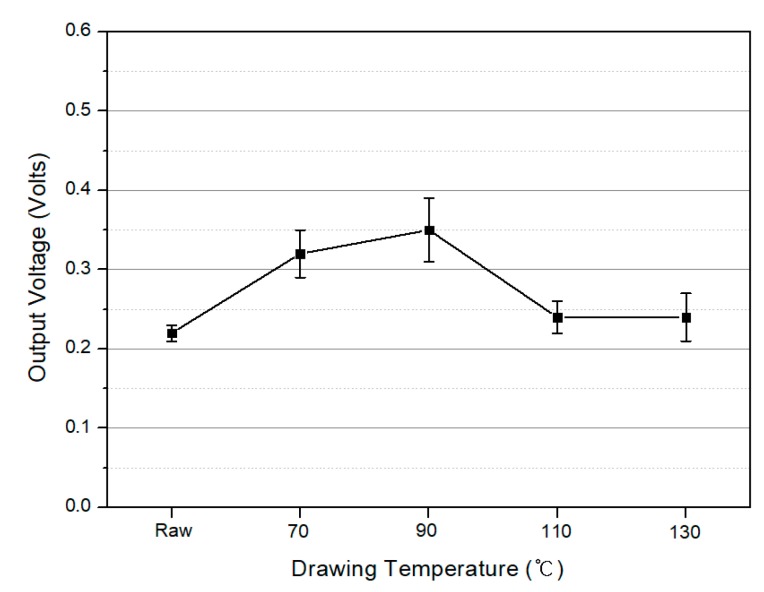
Output voltages of the PVDF/PAR composites as conditions of drawing temperature under a pressure of 30 N (Unpoled).

Figure 13 shows the output voltage obtained when a pressure of 30 N was applied to the PVDF/PAR composites prepared under various drawing conditions. The piezoelectric voltage reached approximately 0.35 V at a drawing temperature of 90 °C. The composite prepared at higher drawing temperatures (110 and 130 °C) showed a decrease in the output voltage (Figure 13).

**Figure 14:**
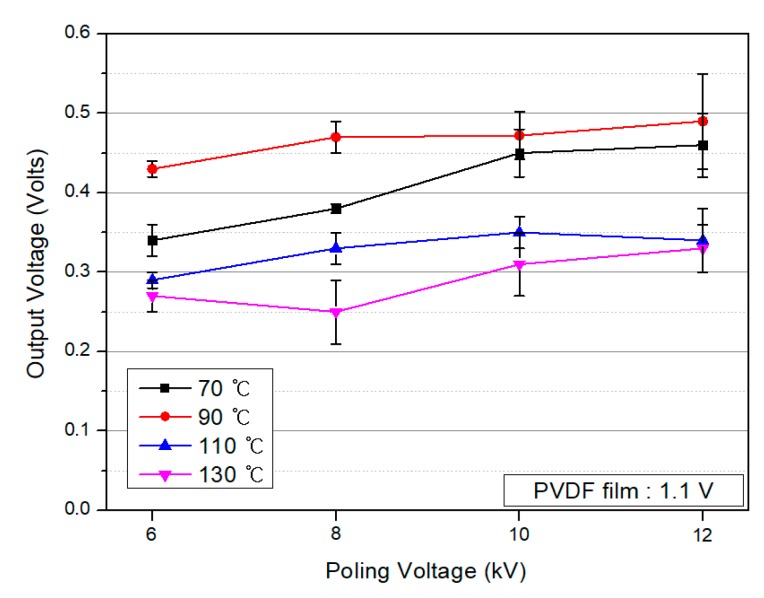
Output voltages of the PVDF/PAR composites as conditions of poling voltage under a pressure of 30 N.

Figure 14 shows the output voltages of the PVDF/PAR composites with poling conditions: it increases with an increase in the poling voltage. Compared to the output voltage of the PVDF film (1.1 V), the PVDF/PAR composites possess almost half of the output value. As described above, because the content of PVDF was only 50% in the composite, the piezoelectricity of the composite material resulted in a 50% lower output voltage. Nevertheless, improvement in the mechanical properties of PVDF composites is important for its structural applications. In the next study, we aim to develop reinforced PVDF composites with a higher output voltage and compromised mechanical properties with a reduced content of the sea component.

**Figure 15:**
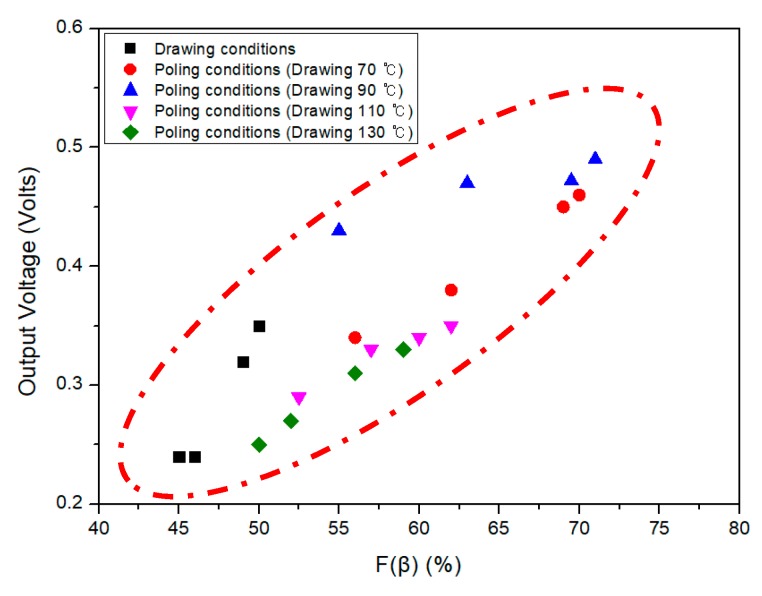
Correlation between *F*(*β*) and output voltage according to various drawing and poling treatments.

Figure 15 shows the relationship between the piezoelectric output voltage and *F*(*β*) of the PVDF/PAR composite. As expected, there is an almost linear correlation; the higher the *F*(*β*) of the composite, the higher the output voltage [20,21].

## 4. Conclusions

In this study, PVDF/PAR island-in-a-sea fibers were prepared by melt conjugate spinning and the PVDF/PAR composites were prepared by compression molding at various molding temperatures. The composites were post-treated by drawing and poling at different drawing temperatures and poling voltages. A molding temperature of 180 °C was identified as the optimal molding temperature for the preparation of PVDF/PAR composites. The *F*(*β)* of the PVDF/PAR composites increased up to a drawing temperature of 90 °C as a result of crystalline phase transition (*α* → *β*) due to the stretching effect. However, at 110 and 130 °C, which are higher than the Curie temperature of PVDF, the *F*(*β)* decreased. In the poling process, *F*(*β)* increased with increasing poling voltage, as the dipoles of the C–F bond of PVDF were aligned in one direction. The highest value of *F*(*β)* was obtained by 12 kV poling of the PVDF/PAR composites drawn at 90 °C. Consequently, the piezoelectric voltage of the PVDF/PAR composites showed the highest value of 0.49 V at a drawing temperature of 90 °C and poling voltage of 12 kV. Therefore, a drawing temperature of 90 °C and a poling voltage of 12 kV were identified as the optimal post-treatment conditions of the PVDF/PAR composites. The *α* crystalline phase of the PVDF component was transformed to the *β* crystalline phase with piezoelectric properties by drawing and poling treatments.

## Figures and Tables

**Table 1:** Spinning conditions of PVDF/PAR island-in-a-sea fiber.

Spinning Conditions	
Sea component	PVDF (Poly(vinylidene fluoride))
Island component	PAR (Polyarylate)
Sea/island ratio	50:50 wt %
Winding speed	650 m/min
